# Short-Term Daily Intake of Polydextrose Fiber Does Not Shorten Intestinal Transit Time in Constipated Adults: A Randomized Controlled Trial

**DOI:** 10.3390/nu10070920

**Published:** 2018-07-19

**Authors:** Peter I. Duncan, Catherine F. Enters-Weijnen, Nashmil Emami, Peter McLean, Tiago Nunes, Maurice Beaumont, Rafael Crabbe, Kevin Whelan, S. Mark Scott, Niek J. deWit, Teunis Weits, Gabriela Bergonzelli, Diederick E. Grobbee

**Affiliations:** 1 Nestlé Research Center, 1000 Lausanne, Switzerland; peter.mclean@takeda.com (P.M.); tiago.alvesnunes@nestle.com (T.N.); gabriela.bergonzelli@rdls.nestle.com (G.B.); 2 Department of Epidemiology, Julius Center for Health Sciences and Primary Care, UMC Utrecht, 3508 GA Utrecht, The Netherlands; c.f.weijnen@umcutrecht.nl (C.F.E.-W.); d.e.grobbee@umcutrecht.nl (D.E.G.); 3 Department of Science, Julius Clinical, 3703 CD Zeist, The Netherlands; 4 Clinical Development Unit, Nestlé Research Center, 1000 Lausanne, Switzerland; nashmil.emami@rdls.nestle.com (N.E.); maurice.beaumont@rdls.nestle.com (M.B.); raf.crabbe57@gmail.com (R.C.); 5 Department of Nutritional Sciences, King’s College London, London SE1 9NH, UK; kevin.whelan@kcl.ac.uk; 6 The Centre for Trauma and Surgery and GI Physiology Unit, The Blizard Institute, Barts and the London School of Medicine and Dentistry, Queen Mary University of London, London E1 1BB, UK; m.scott@qmul.ac.uk; 7 Department of Primary Care, Julius Center for Health Sciences and Primary Care, UMC Utrecht, 3508 GA Utrecht, The Netherlands; n.j.dewit@umcutrecht.nl; 8 Department of Radiology, Diakonessenhuis Utrecht, 3582 KE Utrecht, The Netherlands; teunisweits@gmail.com

**Keywords:** constipation, fiber, polydextrose, adult, randomized controlled trial, intestinal transit, intestinal function, patient-reported outcome

## Abstract

Chronic constipation (CC) remains a common gastrointestinal (GI) disorder that conveys a substantial healthcare burden. Expert guidelines recommend increasing fiber intake, yet the clinical evidence to support this needs strengthening for specific fibers. The aim was to evaluate changes in intestinal transit time and GI symptoms in CC patients who consumed polydextrose. In a randomized, double-blind, placebo-controlled trial, 128 adults with CC received 8 g or 12 g polydextrose, or placebo, daily for 4 weeks. Transit time, as primary outcome, was assessed by radiopaque marker distribution after 2-weeks intervention. Bowel habits, GI symptoms and quality of life (QOL) were assessed by questionnaire, including the Patient-Assessment of Constipation (PAC) Symptoms (SYM), and PAC-QOL. Following 2-weeks intervention, no reduction was seen in transit time in any group and following 2- or 4-weeks intervention, no improvements were seen in stool frequency or consistency in any group. After 2-weeks intervention with 8 g/day polydextrose an improvement was seen in the PAC-SYM rectal score (*p* = 0.041). After 4-weeks intervention both rectal (*p* = 0.049) and stool (*p* = 0.029) scores improved while improvement in the QOL satisfaction score did not reach significance (*p* = 0.071). Overall, the results suggest that 2-weeks consumption of 8 or 12 g/day polydextrose does not significantly improve physiological measures of gut function in CC adults. Longer term consumption may improve clinical measures, but further studies will be required to substantiate this.

## 1. Introduction

Chronic constipation (CC) is a common, heterogeneous, symptom-based GI disorder, with a global pooled prevalence in adults of 14–16%, though prevalence rates vary considerably depending on defining criteria [1,2]. In the absence of an organic cause, characteristic symptoms of CC include infrequent and difficult defecation, feeling of incomplete evacuation, passage of hard stools, and associated abdominal discomfort and bloating [3,4,5]; there is considerable overlap with constipation-predominant irritable bowel syndrome [2,6,7,8]. The prevalence of constipation is higher in females than in males and increases with advancing age in both [1,2,9,10,11]. Pathophysiology of CC is multifactorial and incompletely understood. Nevertheless, it can be broadly considered an overlap between GI/colonic dysmotility (manifested as delayed gut or colonic transit) and impairment of rectal evacuation/pelvic floor dysfunction, which can be due to several identified structural or ‘functional’ obstructive causes [3,12]. The importance of abnormal colorectal sensation [13] and gut dysbiosis [14] are also gaining increasing recognition.

Only a minority of sufferers with CC seek medical help for their condition [15]. However, due to its prevalence, CC still conveys a major healthcare burden. In the US in 2010, CC was the fourth most common GI diagnosis made in patients attending GI clinics, with 3.7 million patient visits estimated [16]. In the UK, annual expenditure on laxatives exceeds £80 million, with 17.4 million laxative medications prescribed in 2012 [17]. It is estimated that 10% of community nursing time is spent on constipation [18]. Perhaps not surprisingly, CC significantly and negatively impacts health-related quality of life (QOL) [19,20,21]. While QOL can be improved by reducing symptoms of constipation [22,23], many patients remain dissatisfied with current treatments [4,24,25], due to variable efficacy, adverse events, cost, taste and inconvenience.

Management of constipation is challenging. Initial recommendations include lifestyle (e.g., increasing fluid intake and levels of exercise) and dietary changes. If these fail, laxatives and other pharmacological approaches are indicated. In general, first-line management, as endorsed by British, American, European and other global guidelines, as well as expert commentaries, is to increase fiber intake through the diet or as a supplement [7,26,27,28,29,30], with 25–35 g/day typically recommended worldwide [31]. Recent systematic reviews and one meta-analysis, combining thirteen trials and nine different fibers, indicate that fiber supplementation is indeed of benefit to patients with CC, with significant increase in stool frequency and softening of stool consistency demonstrated in comparison to placebo [32,33,34]. Nevertheless, further clinical studies are needed to better document the beneficial effect of individual fibers.

Polydextrose (PDX) is a synthetic, randomly bonded polymer, comprising approximately 90% glucose and 10% sorbitol [35] and is considered as a soluble, non-absorbed and partially fermentable fiber. Several studies have tested the effect of PDX (8–30 g/day) on bowel function, with variable results [36,37]. Increases in stool consistency [38,39,40,41,42,43,44] and bulk [43,45,46,47,48] have most frequently been reported. Increased stool frequency has also been documented in several studies [40,41,43,48], though this finding is not consistent [38,42,44,46]. Likewise, PDX has been shown to reduce gut transit time in some studies [39], but not all [43,44,45]. This variability in effect may be due to heterogeneity of study design, populations recruited, and PDX dosage. Indeed, the majority of these studies were performed in ostensibly healthy subjects [43,44,45], with only two studies, by Matsuike et al. [40,41], and a subgroup analysis in a trial by Hengst et al. [39] addressing a target population of constipated adults.

Accordingly, to generate further evidence for the beneficial effect of PDX, the objective of this study was to evaluate changes in physiological (gut transit time) and clinical (GI symptoms) measures of gut function in patients with chronic constipation.

## 2. Materials and Methods

### 2.1. Study Design

This was a randomized, double-blind, placebo-controlled, parallel study comparing three study arms with equal allocation ratio. Participants completed a 2-week run-in phase followed by a 4-week intervention phase. The study followed the principles of the Declaration of Helsinki for research involving human subjects [49] and Good Clinical Practice requirements. It was approved by an independent ethical committee (United Committees on Research Involving Human Subjects (The Netherlands; judgement NL49753.100.14)) and conducted in The Netherlands by Julius Clinical (Zeist, The Netherlands). Written informed consent was obtained from all participants. The study was conducted between October 2014 and November 2015 and was registered prior to participant recruitment at ClinicalTrials.gov as NCT02234518. For reporting, the CONSORT guidelines were followed (see Supplementary Table S1 for the information checklist).

### 2.2. Participants

Participants were recruited from an ad hoc database of patients compiled from a network of approximately 200 general practitioners. Study visits were performed at a single site that housed the X-ray facility. For inclusion, participants were required to be aged 18–75 year, with BMI 18.5–29.9 kg/m^2^, and to fulfil the following retrospectively measured criteria: fiber intake ≤ 18 g/day (determined using the Block Fiber Screener [50], Cleveland Clinic Constipation Score (CCCS) 8–20, and criteria for constipation with the following definition: symptoms of constipation for ≥3 months, mean stool type (consistency) of 1–3 measured using the Bristol Stool Form Scale (BSFS) [51], spontaneous bowel movements 1–3/week, plus at least one of the following: straining on at least 25% of defecations, sensation of incomplete evacuation on at least 25% of defecations, sensation of anorectal obstruction/blockage on at least 25% of defecations or use of manual maneuvers on at least 25% of defecations. Exclusion criteria included pregnant and breast-feeding women, diagnosed GI diseases or complications other than chronic constipation (e.g., Crohn’s disease, celiac disease, IBS, chronic diarrhea), chronic diseases (e.g., cardiovascular, neurological, renal) or medication likely to affect GI motility or limit normal functions (e.g., reduced mobility, increased fragility), and regular consumption of fiber supplements.

### 2.3. Intervention

128 participants were randomized into three groups stratified by gender and females by menstrual cycle phase. During the 2-week run-in period, participants were instructed to continue with their usual diet and physical activity but to discontinue use of products containing probiotics or prebiotics and fiber supplements. Following run-in, participants undertook a 4-week intervention with either polydextrose (Litesse Ultra, Danisco) at 4 g/serving or 6 g/serving, consumed twice daily, for a total supplementation of 8 g/day (low dose) and 12 g/day (high dose), respectively, or placebo (maltodextrin), also provided twice daily. Study products were prepared by Nestlé Product Technology Center (Switzerland) in a milk powder-based format and provided as individual pre-weighed 24 g portions. Each portion was to be mixed with 200 mL water at room temperature and consumed immediately. Total carbohydrate mass content was maintained similar for all groups by varying the maltodextrin added. 

Compliance was determined by daily questionnaire recording consumption, with the threshold determined a priori. Subjects were scored as non-compliant if there was either (1) no consumption of both servings for more than 2 consecutive days during the first 2 weeks of consumption period, (2) no consumption of both servings for more than 3 consecutive days during the latter 2 weeks of the consumption period, or (3) no consumption of both servings of the product on all 3 days prior to a visit.

### 2.4. Outcomes

**Figure 1:**
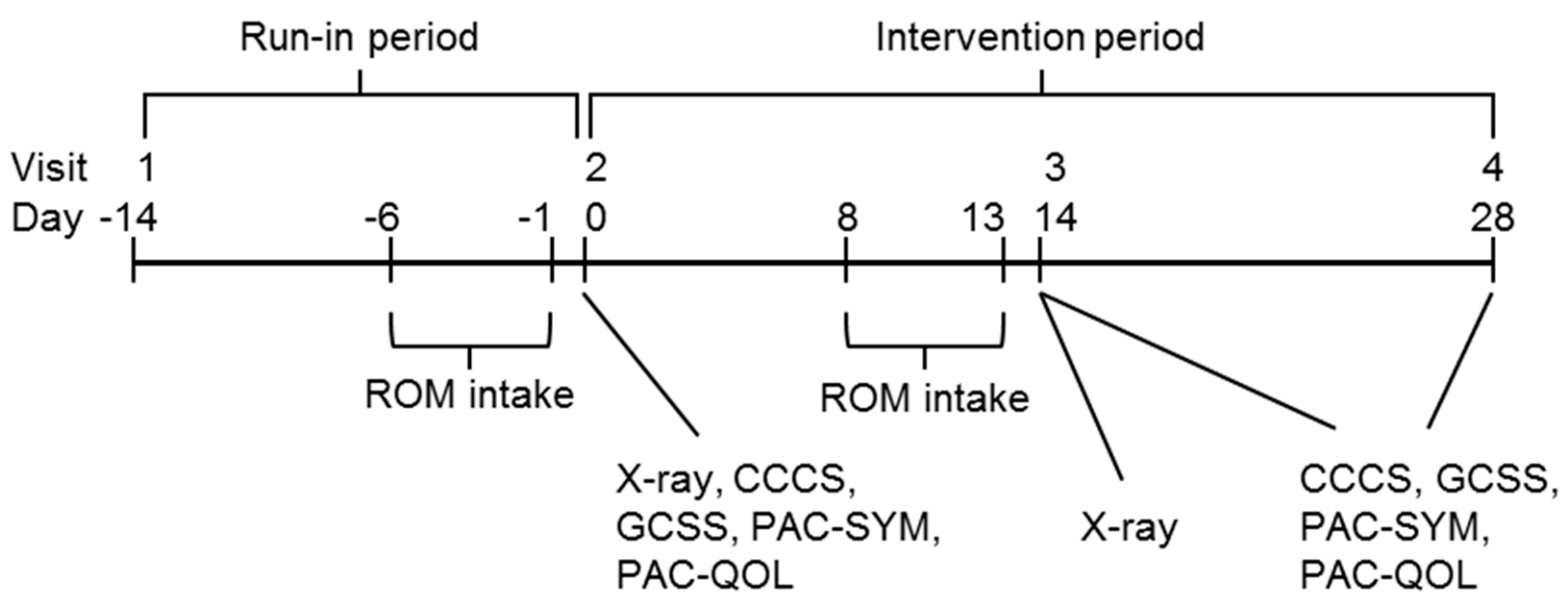
Study activities. ROM, radio-opaque marker; CCCS, Cleveland Clinic constipation score; GCSS, global constipation symptom score; PAC-SYM, patient assessment of constipation—Symptoms; PAC-QOL, PAC—Quality of life.

The primary endpoint was the change in whole GI transit time (WGTT) from baseline (day 0) to the mid-consumption period time point (day 14), as measured by radio-opaque marker (ROM) technique, in the subjects who consumed 12 g/day dose polydextrose compared to those who consumed the placebo. Briefly, participants ingested twelve ROM (CT Transit, Prodimed, France) each day for six consecutive days followed by an abdominal X-ray on the seventh day. Abdominal X-rays were taken on day 0 and day 14 (Figure 1) and WGTT was calculated as previously described [52,53].

Secondary endpoints were determined a priori and included the assessment of changes in WGTT in subjects consuming 8 g/day dose polydextrose compared to placebo, changes in regional colonic transit times (right colon, left colon, and rectosigmoid) and changes in GI symptoms. 

For symptom collection, participants were requested to complete a daily bowel diary in which they recorded for each bowel movement its completeness, spontaneity and consistency (using the BSFS [51]) and the time spent on the toilet, in addition to laxative use in the past 24 h. At baseline (day 0), day 14 and day 28 participants also completed a series of self-reported questionnaires that were used to evaluate symptoms of constipation and QOL. 

Symptom severity was measured using the Patient Assessment of Constipation (PAC) Symptoms (PAC-SYM) questionnaire over a two week recall period, which comprises a twelve-question global score (with each item a 5-point Likert scale from 0, absent to 4, very severe) that can be subdivided into three subdomain scores (abdominal, rectal and stool). Global and subdomain scores are calculated as the mean score of all questions, or the individual subdomain questions, respectively [54]. The global score has been previously used to categories symptoms as mild (score ≤ 1), moderate (>1–2), severe (>2–3) and very severe (>3–4) symptoms [23]. A reduction of 0.6 point on the global score has been suggested as the minimal clinically important difference (MCID) [55]. Symptom severity was also measured using the CCCS, which is based on an eight-item Likert-like scale (score 0, less severe; 30, most severe). A score of >8 indicates constipation, and >15 indicates at least moderate severity of symptoms [56]. 

Global assessment of efficacy was determined by a global constipation symptom score (GCSS) that queried the severity of constipation symptoms relative to the start of the study and was assessed through a single 7-point Likert-like item (markedly worse (−3), somewhat worse (−2), a little bit worse (−1), no change (0), a little better (1), somewhat better (2), markedly better (3)). Abdominal bloating was assessed using a visual analogue scale (from 0 = not at all to 10 = all the time). Health-related QOL was assessed through the PAC-QOL questionnaire [57], which contains 28 questions (5-point Likert scale (0, absent; 4, very severe) grouped into four subdomains (worries and concerns, physical discomfort, psychosocial discomfort, and satisfaction). For analysis, the score of each of the five positively worded questions (No. 18, 25–28) was reverse coded to be consistent with the remaining negatively worded questions. A reduction of 0.5 point on the global score has been suggested as the MCID [57].

Tolerance to the study products was assessed six days after starting the intervention and at the end of the intervention through an 8-item Likert-like scale (assessing abdominal discomfort and distension/bloating, flatulence and nausea over the past 24 h and past 6 days). 

Adverse events were recorded continuously from the signature date of informed consent to 30 days after the last product intake.

### 2.5. Sample Size, Randomization and Blinding

Based on published data and using an expected change in WGTT of 20 h, standard deviation of 29.7 h [58] and setting α = 0.05 and β = 0.20, a sample size of 35 subjects per group was calculated.

Subjects were randomly assigned into the three groups with an equal allocation ratio and incorporating stratification by gender (male/female) and for females by menstrual cycle phase at screening (mid-follicular/mid-luteal phase and post-menopausal). The randomization sequence was determined using Medidata Balance (Medidata Solutions, Inc., New York, NY, USA.)

The test products containing placebo or fibre ingredient were indistinguishable with regard to their sensory properties and were individually coded. Participants, research personnel and those involved in data handling were blinded until locking of the recorded data in a database.

### 2.6. Statistics

The primary endpoint, change from baseline of WGTT, was assessed by an analysis of covariance (ANCOVA) model, correcting for gender and menstrual cycle phase at screening, WGTT at baseline and treatment group. The secondary endpoints were stool frequency (SBM and CSBM), stool consistency (BSFS), bloating, GI tolerance, PAC-SYM, PAC-QOL, CCCS and GCSS, which were similarly analysed by an ANCOVA model correcting for gender and menstrual cycle phase at screening, the symptom baseline value and treatment group. For stool frequency and consistency, weekly averages were calculated from all the values accrued in the week preceding the day of interest.

For the safety analysis, all randomized participants who consumed at least one dose of study product were included. All other analyses were performed using the intention-to-treat population and tests were two-sided with *p* < 0.05 considered statistically significant. Statistical tests were performed using SAS (version 9.2; SAS Institute, Inc., Cary, NC, USA.)

## 3. Results

### 3.1. Participant Recruitment and Study Flow

**Figure 2:**
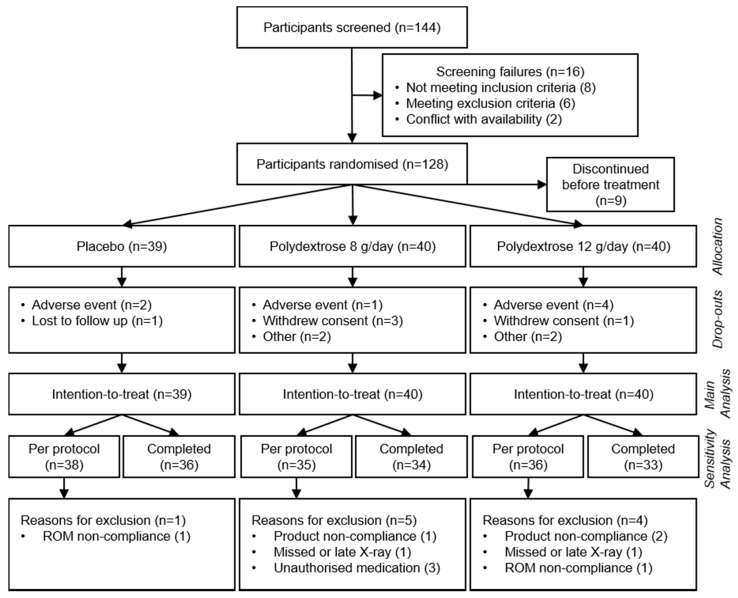
CONSORT participant flow diagram.

The study was conducted between October 2014 and November 2015. Of the 144 individuals who gave informed consent and were screened, 128 were enrolled and randomized into the three treatment groups (polydextrose 8 and 12 g/day, and placebo). Nine subjects dropped out prior to receiving a test product and were thus excluded from the analysis. Of those, eight withdrew with an explanation (i.e., adverse event (1), incomplete ROM intake (3), conflict with visit schedule (2), personal reason (1), and withdrawal of consent (1)) and one withdrew without an explanation. The remaining 119 who received test products were all included in the intention-to-treat analysis (Figure 2). Of the total randomized, 109 participants (85%) completed the first two weeks of intervention and 103 participants (80%) completed the planned four-week intervention. Baseline demographic and clinical characteristics are provided in Table 1.

### 3.2. Primary Endpoint

**Figure 3:**
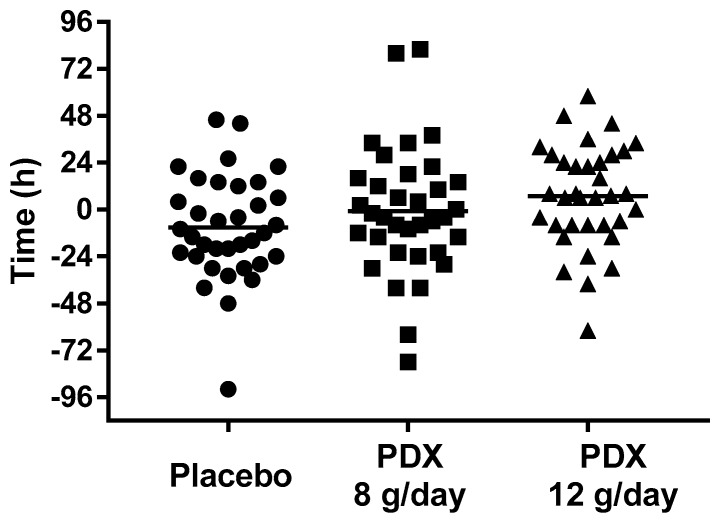
Change in whole gut transit time after two weeks intervention with polydextrose (PDX). Each point represents a study participant (ITT population). Bar indicates the mean.

Baseline WGTT values were not different between treatment groups (Table 1). Following two weeks intervention the 12 g/day dose treatment group did not experience a statistically significant change in WGTT from baseline compared to placebo (mean difference 11.8, 95% CI −1.3, 24.9, *p* = 0.077) (Figure 3 and Table 2).

### 3.3. Secondary Endpoints

Analysis of the 8 g/day dose treatment group revealed no significant change in WGTT compared to placebo (Table 2; mean difference 6.7, 95% CI −6.3, 19.6, *p* = 0.309). In all groups a restricted number of subjects had very short WGTT. Exclusion of those subjects did not change the observed outcome effect on WGTT of either 8 g/day or 12 g/day polydextrose (data not shown).

Regional colon transit times suggested that the change seen in WGTT with the 12 g/day dose polydextrose compared to the placebo was accounted for by the left colon segment (Table 2; mean difference 9.3, 95%CI 2.2–16.5, *p* = 0.01). No difference in the left colon transit was seen in the 8 g/day dose polydextrose group compared to placebo (mean difference 1.7, 95% CI −5.4–8.8, *p* = 0.633). In addition, no differences were seen between the treatment groups in either the right colon or rectosigmoid regions.

Changes in the frequency of spontaneous bowel movements (SBM) (based on data collected during the week leading up to the study site visit) were similar between groups after both 14 days and 28 days of intervention (Table 2). When the frequency of complete spontaneous bowel movements (CSBM) was assessed the 12 g/day dose polydextrose group had a trend to reduced CSBM after 14 days intervention compared to the placebo group (Table 2). This trend was not maintained after 28 days of intervention and was not observed with the 8 g/day dose polydextrose group at either time point (data not shown). No differences between groups were seen in stool consistency at either time point (Table 2 and data not shown). 

By sensitivity analysis, when the full data set was used (i.e., using all data collected between visits), an improvement in SBM stool frequency was seen between the 8 g/day dose group and the placebo (mean difference 1.1, 95% CI 0.1–2.2, *p* = 0.030). No differences were seen with the 12 g/day dose group. Additionally, with the full data set, no differences were seen between groups in stool consistency at either time point (data not shown).

### 3.4. Patient-Reported Symptoms

The mean PAC-SYM global scores at baseline were 1.7 (SD 0.6), 2.0 (SD 0.7) and 1.6 (SD 0.5) for the placebo, 8 g/day dose polydextrose and 12 g/day dose polydextrose groups, respectively, with significant differences seen between the groups for the global score (8 g/day polydextrose vs. placebo, *p* = 0.018) and for the abdominal subdomain score (8 g/day polydextrose vs. placebo, *p* = 0.039). Following two weeks intervention, the change from baseline of the global score in the 8 g/day dose polydextrose group compared to the placebo showed a numerical trend towards improvement, which after 4-weeks intervention was significantly different (Table 3). The 12 g/day dose group did not show this improvement.

Within the rectal subdomain, the 8 g/day dose group had a significant improvement compared to placebo at both 2- and 4-weeks treatment. In the 12 g/day dose group a difference was also seen but remained a trend at 14 days (*p* = 0.053) and 28 days (*p* = 0.082). An improvement in the stool subdomain score was seen after 4-weeks intervention with 8 g/day dose polydextrose compared to the placebo. No differences were seen for either group in the abdominal subscore.

Assessment of symptoms with the CCCS questionnaire did not reveal any change from baseline of the total score in any group (data not shown).

Finally, non-statistically significant improvements were detected after 4-weeks intervention with the 8 g/day dose polydextrose compared to the placebo in bloating, global and satisfaction subdomain QOL scores and when the overall severity of symptoms was queried with the GCSS (Table 4).

**Figure 4:**
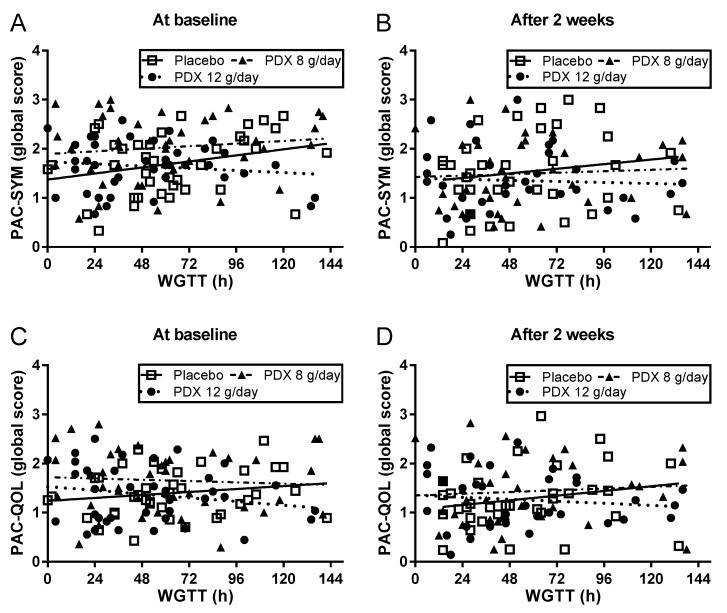
Regression analysis of whole gut intestinal transit time (WGTT) and global scores of PAC-SYM (**A**,**B**) and PAC-QOL (**C**,**D**) at baseline (**A**,**C**) and after two weeks intervention of polydextrose (PDX) (**B**,**D**). Individual data points with regression lines are shown.

Whether relationships exist between WGTT and patient-reported outcomes was also assessed. As shown in Figure 4 no relationship was found between WGTT and either the global score of PAC-SYM or PAC-QOL at either baseline or following 2-weeks intervention.

### 3.5. Adverse Events

Product tolerance was assessed by questionnaire after six and 28 days of intervention. No changes from baseline were seen in any group (data not shown).

No association was seen between any treatment group and the occurrence of adverse events (Table 5) nor between the treatment group and the study product (*p* = 0.291, Fisher’s exact test).

## 4. Discussion

The positive impact that consumption of polydextrose has been reported to have on bowel function [36,37] led us to hypothesize that 8–12 g/day of polydextrose would lead to a reduction in WGTT and improve symptoms of constipation in adults. The lower dose of 8 g/day was chosen based on doses (7–8 g/day) reported at the time to have an impact on bowel function in constipated participants [39,40,41]. While both lower (4 g/day) and higher (12 g/day) doses were also reported to have an impact on bowel function in healthy subjects [48], a single higher dose of 12 g/day was tested here in the context of chronic constipation. The results of this study, however, do not satisfy the primary objective as no statistically significant change in WGTT was found between those who consumed 12 g/day polydextrose and those who consumed placebo for 2-weeks. Indeed, rather than a reduction in WGTT, the tendency of the 12 g/day dose intervention was to lengthen the WGTT. Secondary outcome analyses suggested that this lengthening was accounted for by the left colon, and although there was no change in the number of SBMs, it may also have led to a reduction in the number of CSBMs. The frequency of CSBM has been suggested to be a better outcome measure than SBM, given that it reflects the subjective completeness of a defecation event. While transit time was not measured at the end of the 28 day intervention period, this change in CSBM relative to the placebo was no longer detected at this time. In this study, the lower dose of polydextrose (8 g/day) also did not show a significant change in WGTT or a change in stool frequency or consistency.

A major challenge when studying chronic constipation is the inter-individual heterogeneity in what can be considered as “normal” for stool frequency, consistency and the range of accompanying symptoms. Added to this is the challenge that, between individuals, symptoms may be interpreted differently, and their most bothersome symptom or feature can differ largely. Given the inconsistencies of effects reported with polydextrose in the published literature on stool frequency, stool consistency and intestinal transit (endpoints that may be considered as key diagnostic criteria for constipation), we thought it important to assess symptoms and QOL as patient-reported outcomes. Using a selection of these assessment instruments, the only significant change seen that also met the MCID change criterion [55] was the PAC-SYM stool subscore after 4-weeks intervention of the low dose of polydextrose (8 g/day). The absence of a significant increase in GCSS is consistent with a general lack of a significant clinical effect for polydextrose under these conditions.

That this study was unsuccessful may feasibly be due to subject heterogeneity in the most bothersome symptom. We used modified Rome III diagnostic criteria for chronic functional constipation as inclusion conditions, and to limit the severity to those with mild to moderate constipation a CCCS score of 8–20 was set, as used elsewhere [59,60,61]. At baseline, the groups were well balanced, although compared to the placebo group, the 12 g/day dose polydextrose group had a slightly higher, albeit statistically non-significant, age (median 47 year vs. placebo 31 year, *p* = 0.11) and slightly lower daily intake of fiber (14.2 vs. placebo 15.3 g/day, *p* = 0.052). Nevertheless following the run-in period during which daily bowel diary records were kept, many subjects reported higher stool frequency (median stool frequency (4.0–5.0)) than the inclusion criterion of 1–3 movements per week. The corresponding stool consistency values remained within the inclusion criterion value of ≤3, although given the variability seen, some participants clearly had a score above this (median stool consistency (2.7–3.2)). The results of a sensitivity analysis of those participants fulfilling the inclusion criteria for both stool frequency and consistency at baseline were consistent with that of the full data set for changes in WGTT, stool frequency and stool consistency (data not shown). The failure to maintain certain elements of the inclusion criteria post-screening is not uncommon in functional GI disorders and indeed the potential for symptom variability is encompassed within the Rome III criteria. Clearly, further studies powered to detect changes and relief of patient-reported symptoms will be needed. Furthermore, while we tested two doses of polydextrose it may be that these were too high. As a dose of 4 g/day has already been reported to have a positive impact on bowel function in healthy subjects [48], additional dose range finding studies in constipated individuals may also be beneficial.

The published literature on the impact of polydextrose has primarily focused on healthy adults and thus even if the subjects in this trial were not constipated we may have expected to see a positive impact on WGTT, stool frequency or consistency. But the impact in the healthy individual is not conclusive and in a recent scientific opinion, the European Food Safety Authority ruled that the evidence was insufficient to demonstrate an impact of polydextrose in the maintenance of normal defecation [62]. Furthermore, the quantity of evidence in constipated adults is low. Two non-placebo-controlled studies suggested that polydextrose could improve stool frequency and consistency after 10 days intervention [40,41]. Meanwhile another study demonstrated that intestinal transit time decreased with polydextrose [39]. The authors reported that this improvement, and that of stool consistency, was seen primarily in a constipated subgroup. Unfortunately, the subgroup analysis was not shown, limiting the strength of conclusion that can be drawn from these data. 

The limited positive effects of polydextrose seen in this study and the relative variability in symptom improvement seen in the published literature, including in healthy subjects, suggest that polydextrose may not be the ideal fiber to benefit a heterogeneous population suffering from constipation. Polydextrose has been reported to be slowly fermented in the colon and early reports suggested that approximately 30–50% of the ingested dose is excreted [45,63]. Incomplete fermentation should prolong its water binding and stool bulking capacity, thus improving stool consistency and volume, two properties believed to facilitate defecation [64]. However, a more recent report estimated that only 10% of the ingested dose of polydextrose was excreted [38]. Such large differences in the amount excreted support the notion that subject variances in the fermentative capacity of the colonic microflora exist [37], and that this capacity may determine the beneficial effect of polydextrose. Therefore, individuals who ferment polydextrose more completely may thus not benefit from its defecation-improving effect [65]. Constipation has been associated with changes in the colonic microbiota [66,67,68] but an association of microbiota to constipation symptom response with polydextrose has yet to be shown.

This study has several strengths, not least that it queried a comprehensive array of constipation-related symptoms and quality of life factors using condition-specific instruments and tested two doses of polydextrose. In addition, the randomized and blinded allocation and use of placebo aimed to minimize bias in testing our hypothesis. 

The study also has several limitations. Baseline values of key constipation symptoms did not all meet the inclusion criteria that were measured during screening. Re-screening of participants at baseline and prior to intervention may be a necessary step [69]. Additionally, slow WGTT was not used as a screening/inclusion criterion, which may have improved subject homogeneity and thus response to intervention [69,70,71]. Further to this, a change in WGTT was only evaluated at 2-weeks whereas the patient-report outcome effects suggest that at least 4-weeks intervention may be required. Also, although the intake of dietary fiber was assessed at screening as an inclusion criterion and participants were instructed to maintain their regular diet during the study, it was not assessed following the intervention. Finally, we do not see it as a weakness that our study had a majority of female participants. We did not fix a women:men ratio for the study and although the data we present is mostly in women it broadly reflects the reported sex differences in prevalence, and therefore actually increases the external validity to the population we are investigating. However, gender balance may need to be considered as it has been reported that there may be gender differences in specific symptoms of constipation [9] and likely also pathophysiology [72,73]. Women represented >90% of the participants, which precluded a sensitivity analysis to assess if these results are translatable to men.

In conclusion, this is the first randomized clinical trial to test the impact of polydextrose in chronically constipated adults. Consumption of 8 g/day or 12 g/day polydextrose for 2 weeks did not improve measures of intestinal transit, stool frequency or stool consistency. However, our results suggest that future studies in constipated subjects with longer term intervention (i.e., 4 weeks or longer) and focused on rectal and stool-related symptoms as well as on QOL-related satisfaction may be more insightful.

## Figures and Tables

**Table 1:** Baseline population characteristics (ITT population).

	Placebo (*n* = 39)	Polydextrose 8 g/day (*n* = 40)	Polydextrose 12 g/day (*n* = 40)
**Age (years), median (range, SD)**	30.9 (19–71, 16.0)	29.2 (19–72, 15.7)	46.8 (19–64, 15.1)
**Female, *n* (%)**	36 (92.3)	38 (95.0)	37 (92.5)
**BMI (kg/m^2^), mean (SD)**	23.9 (3.3)	23.6 (2.9)	23.7 (2.8)
**Fiber intake, g/day (SD)**	15.3 (2.4)	15.2 (2.3)	14.2 (2.5)
**CCCS, mean (SD)**	12.8 (3.3)	13.6 (2.6)	12.4 (2.7)
**SBM (*n*/week), mean (SD)**	5.4 (3.0)	4.9 (3.9)	6.3 (4.4)
**BSFS, mean (SD)**	2.8 (1.1)	2.8 (1.1)	3.0 (1.2)
**Bloating, mean (SD)**	5.5 (2.4)	6.4 (2.3)	6.0 (2.2)
**PAC-SYM (global), mean (SD)**	1.7 (0.6)	2.0 (0.7) *	1.6 (0.5)
**PAC-QOL (global), mean (SD)**	1.4 (0.5)	1.7 (0.6) *	1.3 (0.6)
**WGTT (hours), mean (SD)**	64 (35)	62 (41)	51 (34)

BMI, body mass index; CCCS, Cleveland Clinic constipation score; SBM, spontaneous bowel movements per week; BSFS, Bristol stool form scale (weekly mean); PAC-SYM/QOL, patient assessment of constipation-symptoms/quality of life; WGTT, whole gut transit time; SD, standard deviation. * *p* < 0.05 for comparison vs. placebo.

**Table 2:** Whole gut transit time, regional colon transit time, stool frequency and stool consistency changes from baseline (ITT population).

	Placebo		Polydextrose 8 g/day		Polydextrose 12 g/day		Change from Baseline. Polydextrose 12 g/day vs. PLACEBO (ANCOVA Model)	
	*n*	Mean ± SD	*n*	Mean ± SD	*n*	Mean ± SD	Mean Difference (95% CI)	*p*-Value
Change in WGTT (h)								
	35	−9.4 ± 27.0	35	−0.9 ± 32.8	35	6.8 ± 26.1	11.8 (−1.3, 24.9)	0.08
Change in Regional CTT (h)								
Right colon	35	−4.4 ± 17.1	35	0.5 ± 13.7	35	−1.3 ± 11.6	0.5 (−5.2, 6.2)	0.86
Left colon	35	−1.6 ± 12.8	35	0.4 ± 18.5	35	9.4 ± 16.1	9.3 (2.2, 16.5)	0.01 *
Rectosigmoid	35	−2.7 ± 16.5	35	−1.7 ± 18.7	35	−0.7 ± 14.0	−0.7 (−7.0, 5.6)	0.83
Change in Stool Frequency (Number Per Week)								
SBM, 14 days	36	0.6 ± 2.9	35	1.3 ± 3.3	36	0.7 ± 3.3	0.2 (−1.2, 1.7)	0.77
SBM, 28 days	36	0.1 ± 2.7	34	0.8 ± 2.6	34	−0.6 ± 3.3	−0.4 (−1.6, 0.8)	0.52
CSBM, 14 days	36	1.4 ± 2.6	35	1.1 ± 2.1	36	0.1 ± 2.1	−1.0 (−2.1, 0.0)	0.06
CSBM, 28 days	36	0.6 ± 2.1	34	1.0 ± 2.0	34	−0.2 ± 2.3	−0.5 (−1.5, 0.5)	0.29
Change in Stool Consistency								
BSFS, 14 days	36	0.3 ± 1.4	34	0.2 ± 1.2	36	0.3 ± 1.1	0.2 (−0.3, 0.7)	0.51
BSFS, 28 days	36	0.1 ± 1.2	34	0.3 ± 1.1	34	0.3 ± 1.2	0.4 (−0.1, 0.8)	0.14

SBM, spontaneous bowel movements; CSBM, complete SBM; BSFS, Bristol stool form scale (1 = separate hard lumps, 7 = entirely liquid); SD, standard deviation; CI, confidence interval. * *p* < 0.05.

**Table 3:** PAC-SYM change from baseline differences (ITT population).

	Placebo		Polydextrose 8 g/day		Polydextrose 12 g/day	
	*n*	Mean ± SD	*n*	Mean ± SD	*n*	Mean ± SD
Change in Global Score						
14 days	36	−0.2 ± 0.6	35	−0.5 ± 0.7 *	36	−0.2 ± 0.6
28 days	36	−0.2 ± 0.5	34	−0.7 ± 0.6 **	34	−0.4 ± 0.8
Change in Abdominal Score						
14 days	36	−0.3 ± 0.7	35	−0.6 ± 0.8	36	−0.2 ± 0.7
28 days	36	−0.3 ± 0.8	34	−0.8 ± 0.7	34	−0.3 ± 0.9
Change in Rectal Score						
14 days	36	0.1 ± 0.6	35	−0.4 ± 0.8 **	36	−0.2 ± 0.7 *
28 days	36	−0.1 ± 0.4	34	−0.5 ± 0.7 **	34	−0.3 ± 0.8 *
Change in Stool Score						
14 days	36	−0.2 ± 0.8	35	−0.6 ± 1.0	36	−0.3 ± 0.8
28 days	36	−0.2 ± 0.8	34	−0.8 ± 0.9 **	34	−0.4 ± 1.0

PAC-SYM, patient assessment of constipation-symptoms; SD, standard deviation. * *p* < 0.1, ** *p* < 0.05 for comparison vs. placebo and are derived from the ANCOVA modelling.

**Table 4:** Quality of life and symptom changes from baseline after 4-weeks intervention with polydextrose.

	Placebo		Polydextrose 8 g/day		Polydextrose 12 g/day	
	*n*	Mean ± SD	*n*	Mean ± SD	*n*	Mean ± SD
Change in PAC-QOL Global Score						
28 days	36	−0.1 ± 0.5	34	−0.4 ± 0.6 *	34	−0.3 ± 0.7
Change in PAC-QOL Satisfaction Score						
28 days	36	−0.1 ± 0.9	34	−0.6 ± 0.9 *	34	−0.3 ± 1.2
Change in Bloating Score						
28 days	33	0.2 ± 1.9	32	−1.0 ± 2.4 *	33	−0.6 ± 3.1
Change in GCSS						
28 days	36	0.2 ± 1.7	33	0.9 ± 1.8 *	33	0.5 ± 1.9

PAC-QOL, patient assessment of constipation-quality of life; GCSS, global constipation symptom score; SD, standard deviation. * *p* < 0.1 for comparison vs. placebo and are derived from the ANCOVA modelling.

**Table 5:** Adverse events reported by study participants.

	Placebo		Polydextrose 8 g/day		Polydextrose 12 g/day		*p*-Value
	Subjects, *n* (% Group)	Events, *n*	Subjects, *n* (% Group)	Events, *n*	Subjects, *n* (% Group)	Events, *n*	
**Any AE/SAE**	24 (61.5%)	69	29 (72.5%)	71	30 (75.0%)	81	0.39
**Abdominal discomfort**	0 (0%)	0	1 (2.5%)	1	1 (2.5%)	1	1.00
**Abdominal distension**	3 (7.7%)	4	6 (15%)	6	3 (7.5%)	3	0.32
**Abdominal pain**	9 (23.1%)	18	11 (27.5%)	18	8 (20.0%)	12	0.74
**Flatulence**	0 (0%)	0	1 (2.5%)	1	0 (0%)	0	1.00
**Nausea**	2 (5.1%)	2	3 (7.5%)	3	3 (7.5%)	4	1.00

The number of subjects reporting at least one adverse event (AE) or serious adverse event (SAE) and the total number of events per treatment group. Significance was assessed with the Fisher’s exact test.

## Supplementary Materials

The following are available online at http://www.mdpi.com/2072-6643/10/7/920/s1, Table S1: CONSORT checklist.
